# Comparing three complete mitochondrial genomes of the moss genus *Orthotrichum* Hedw.

**DOI:** 10.1080/23802359.2016.1149784

**Published:** 2016-03-31

**Authors:** Beatriz Vigalondo, Yang Liu, Isabel Draper, Francisco Lara, Ricardo Garilleti, Vicente Mazimpaka, Bernard Goffinet

**Affiliations:** a Departamento de Biología (Botánica), Facultad de Ciencias, Universidad Autónoma de Madrid, Madrid, Spain;; b Department of Ecology and Evolutionary Biology, University of Connecticut, Storrs, CT, USA;; c Departamento de Botánica, Facultad de Farmacia, Universidad de Valencia, Burjassot, Spain

**Keywords:** Bryophytes, complete mitogenome, *Orthotrichaceae*

## Abstract

Here, we present a comparative analysis of the mitochondrial genome of three representatives of *Orthotrichum* Hedw (Bryophyta): two populations of *O. diaphanum* and one of the related species, namely *O. macrocephalum*. Their mitochondrial genomes share the same gene content and gene order, and are furthermore structurally identical to those of other arthrodontous mosses. The mitogenome of the allopatric samples of *O. diaphanum* differ in 0.1% of their sequence, with protein coding genes holding five mutations, including two non-synonymous changes. The divergence between the mitogenomes of the two species, *O. diaphanum* and *O. macrocephalum,* is 0.4%. Within a broader sampling of the *Orthotrichaceae*, patterns of genome divergence are consistent with phylogenetic relationships.

The genus *Orthotrichum* is one of the most species-rich moss genera, with ∼163 species (Medina et al. 2013). *Orthotrichum diaphanum* Brid. and *O. macrocephalum* F. Lara, Garilleti and Mazimpaka are two related epiphytic species of section *Diaphana* Vitt. (Lara et al. 1994) with distinct but overlapping geographic distributions: *O. diaphanum* occurs throughout the Western Palearctic–Western Nearctic, whereas *O. macrocephalum* is restricted to the Mediterranean areas in the Northern Hemisphere.

The number of moss mitochondrial (mt) genomes announced has dramatically increased in recent years (Liu et al. 2011, 2014; Sawicki et al. 2014, 2015; Alonso et al. 2015), but only one study (Lewis et al. 2016) has targeted the mt genome of several conspecific populations. We sought to assess the types and distribution of substitutions between the genome from two populations of *O. diaphanum* and between this species and the related *O. macrocephalum*.

Multiple gametophytes and/or sporophytes were collected from three samples: *O. diaphanum* #1 (MAUAM-Brio 4559; Spain, 6°45′34.2″N 5°22′07.3″W), *O. diaphanum #2* (MAUAM-Brio 4560; Germany, 10, 52°18′14.8″N 12°59'11.3''E) and *O. macrocephalum* (MAUAM-Brio 4561; Spain, Hoyo de Manzanares, 40°37′15″N 3°54′48.25″W). Total DNA was extracted using the NucleoSpin plant II^®^ Midi kit (Macherey Nagel GmbH & Co. KG, Düren, Germany). Three genomic DNA libraries were prepared using the Nextera kit (Illumina, CA), and then multiplexed and sequenced on an Illumina MiSeq instrument using a 600-cycle v3 sequencing kit (Illumina, CA). Following the filtering and trimming of the reads with Trimmomatic v0.33 (Bolger et al. 2014), the resulting paired-end reads were *de novo* assembled using CLC Genomics Workbench v6.5 (CLC Bio, Aarhus, Denmark) with the default assembly parameters. All *de novo* contigs were blasted with CLC BLAST tool to the *O. stellatum* Brid. mt genome (NC_024522, Liu et al. 2014). A single mt contig was obtained for *O. diaphanum* #1 (total contigs = 27 499; N50 = 1 444 bp) and *O. diaphanum* #2 (total contigs = 65 946; N50 = 1 773 bp), whereas for *O. macrocephalum* two contigs were recovered (total contigs = 25 937; N50 = 1 869 bp). All contigs were first visually inspected for unexpected drops in depth, and then aligned against the reference and imported to Geneious (Biomatters Ltd., Auckand, New Zealand). Low-depth areas in a contig or gaps between contigs were confirmed or closed through a series of reference alignments and assemblies following Fučíková et al. (2014). These gap sequences were verified by PCR and Sanger sequencing. The complete mt genomes were annotated in Geneious 7.1.2 using extracted annotations from *O. stellatum*. Coding regions were checked with an ExPASy translation tool (Gasteiger et al. 2003), and annotations were manually corrected. Exon and intron boundaries were further confirmed against orthologs from other species.

To confirm the phylogenetic identity of the samples, we inferred their relationships with other 13 moss species publicly available, including members of the *Orthotrichaceae* (see Figure 1 for GenBank accession numbers). Protein-coding genes sequences were aligned using the progressive Mauve algorithm (Darling et al. 2004) in Geneious, in order to perform phylogenetic analyses under maximum likelihood and Bayesian inference.

**Figure 1.**
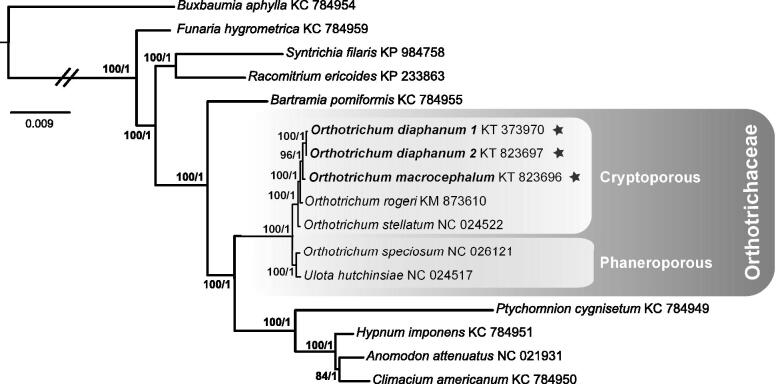
Majority-rule consensus tree of the Bayesian inference analyses of 40 mitochondrial protein coding genes, showing the phylogenomic affinities of *O. diaphanum* and *O. macrocephalum* (indicated with stars). Bootstrap values under maximum likelihood (>50) followed by posterior probabilities (>0.95) of Bayesian inference are indicated near the corresponding branch. GenBank accession numbers follow taxon names. Scale bar represents substitutions per site rate.

The total length for the mt genome of *O. diaphanum #*1 (KT_373970) is 104 756 bp (106× coverage), *O. diaphanum #*2 (KT_823697) 104 744 bp (163× coverage) and *O. macrocephalum* (KT_823696) 104 624 bp (60× coverage). The GC content of the three samples is the same as for other published *Orthotrichaceae* (i.e. 39.8%; Liu et al. 2014; Sawicki et al. 2014, 2015). The three mt genomes contain the same set of genes (i.e. 40 protein-coding, 24 tRNA and 3 rRNA genes) organized in the same order as in other *Orthotrichaceae* and most other mosses (Liu et al. 2014; Sawicki et al. 2014, 2015; Young-Jun et al. 2015).

The phylogenetic inferences (Figure 1) are congruent with the phylogenetic structure among moss genera (Liu et al. 2014; Young-Jun et al. 2015). *Orthotrichum* is known to be polyphyletic, which is confirmed here with species of *Orthotrichum* with superficial stomata more closely related to *Ulota* D. Mohr than to species with immersed stomata (Goffinet et al. 2004).

The two mt genomes of *O. diaphanum* differ in 68 bp (i.e. 0.1%), and when *O. macrocephalum* is added, the number of variable sites increases to 398 bp (i.e. 0.4%). Across *Orthotrichum* species with immersed stomata (cryptoporous; *O. diaphanum, O. macrocephalum, O. rogeri* Brid and *O. stellatum*) the mitogenomes differ in 1 241 bp (i.e. 1.2%), whereas the two taxa with superficial stomata (phaneroporous; *O. speciosum* Nees and *Ulota hutchinsiae* (Sm.) Hammar) differ in 605 bp (i.e. 0.6%). The divergence between species of *Orthotrichum* with immersed and superficial stomata is 1 903 bp (i.e. 1.8%), which is higher than between *O. speciosum* and *Ulota,* as would be expected from their phylogenetic relationship (Goffinet et al. 2004) (Figure 1). Within the *Orthotrichoideae*, the mitogenome varies in 2 288 sites (i.e. 2.1%). Compared to the other moss subfamily for which more than two mitogenomes have been assembled, the *Orthotrichoideae* exhibit more variation than the three species of *Funarioideae* (i.e. 1.5%; Liu et al. 2014).

Within *O. diaphanum*, the variable sites are relatively scarce and widely dispersed along the mt genome. Sixty-three substitutions occur within non-coding regions, and five (three transitions and two transversions) within protein-coding regions. Among the latter, two substitutions result in non-synonymous changes (i.e. in the *rps*1 gene: A<–>C, 3rd codon position of the 211th codon, Asparagine to Lysine; *ccmFN* gene: A<–>G, 1st codon position of the 175th codon, Asparagine to Aspartic acid). The only concentration of mutations occurs in the *cox*1 group II intron *cox1i1064g2*, which holds two mononucleotide substitutions, one 6 bp indel and either five or three TATAT microsatellite repeats in *O. diaphanum #*1 and #2, respectively. The alignment of both *O. diaphanum* and *O. macrocephalum* mitogenomes, and that of all *Orthotrichaceae*, reveals noticeable interspecific variation, most of it in non-coding regions, such as *cox*1 and *cox*2 group II introns, and also within coding regions such as *ccmFN* gene. Those regions could potentially be evaluated as new markers for phylogenetic analyses within this moss family.
